# Supplementary material for: The association of cobalturia with cobaltism symptoms a prospective blinded study of 229 post-arthroplasty patients
Source: PLoS One. 2023 Dec 21;18(12):e0295203. doi: 10.1371/journal.pone.0295203 (PMC10734948; doi:10.1371/journal.pone.0295203)
Supplement: S1 File — (PDF) [file pone.0295203.s003.pdf]

Research &
Graduate Studies
UNIVERSITY of ALASKA ANCHORAGE

3211 Providence Drive
Anchorage, Alaska 99508-4614
T 907.786.1099, F 907.786.1791
www.uaa.alaska.edu/research/ric

DATE:	May 2, 2019
TO:	Stephen Tower, MD
FROM:	University of Alaska Anchorage IRB
PROJECT TITLE:	[1062885-2] Consequences of Corrosion or Mechanical Wear of Chrome-Cobalt-Containing Arthroprosthetic Implants
SUBMISSION TYPE:	New Project
ACTION:	DETERMINATION OF NOT HUMAN SUBJECTS RESEARCH
DECISION DATE:	May 2, 2019

Thank you for your submission of the Not Human Subjects Research Self Determination form for your proposed research study. The University of Alaska Anchorage IRB has determined this project does not meet the definition of human subject research under the purview of the IRB according to federal regulations. Note that prior to making any change to the described protocol please communicate your intentions to the IRB as those changes may invalidate your Not HSR status.

We will retain a copy of this correspondence within our records.

If you have any questions, please contact Robert Boeckmann at (907) 786-1793 or rjboeckmann@alaska.edu. Please include your project title and reference number in all correspondence with this office.

Robert J. Boeckmann, Ph.D.

Chair, Institutional Review Board

Generated on IRBNet
