# Supplementary material for: The association of cobalturia with cobaltism symptoms a prospective blinded study of 229 post-arthroplasty patients
Source: PLoS One. 2023 Dec 21;18(12):e0295203. doi: 10.1371/journal.pone.0295203 (PMC10734948; doi:10.1371/journal.pone.0295203)
Supplement: Attachment — Submitted filename: P1 response to reviewers 230924.docx [file pone.0295203.s004.docx]

PONE-D-23-19885

The association of Cobalturia with Cobaltism Symptoms

A prospective blinded study of 229 post-arthroplasty patients

PLOS ONE

Corresponding Author responds to Reviewers

I appreciate the efforts of the reviewers, edits made to the manuscript will be detailed in red in the context of their critique.

Reviewer #1: The paper needs major revisions. Perform all the suggestions:
Here are some more detailed suggestions for improving each section:

Introduction:

- Provide 2-3 sentences explaining cobaltism - a clinical syndrome caused by cobalt exposure leading to neurological, cardiovascular, and other manifestations. cite PMID: 21066926. See lines 30-38.

- line 48, among differential diagnose other causes should be discussed leading to cognitive impairment, fatigue, sleepiness as obstructive apnea. Please cite doi:10.1007/s11325-021-02520-y. See lines 40-44 and 66-73.

Added references

26.	Asker S, Asker M, Yeltekin AC, Aslan M, Demir H. Serum levels of trace minerals and heavy metals in severe obstructive sleep apnea patients: correlates and clinical implications. Sleep and Breathing. 2015;19:547-52.

- Note that cobalt release from joint implants is a major cause today. See lines 31-38.

Briefly explain mechanisms - corrosion, wear, release into blood/urine. See lines 31-32.
- State that relationship between symptoms, urine cobalt levels, and exposure time is not well studied. See lines 75-79

Methods:

- In developing symptom questionnaire, describe process for choosing domains, consulting existing questionnaires, pilot testing questions. See lines 87-93.
- For urine cobalt testing, explain choice of 1 ppb cutoff based on prior studies showing associations with clinical effects at this level. See lines 114-129.
- Describe all statistical tests used - correlations, comparisons between groups, ROC analysis. See lines 134-137, I believe ROC analysis is not appropriate for this study because there is no gold standard for the diagnosis of orthopedic-implant cobaltism, additionally the quartile analysis for both spot urine [Co] and the product of urine [Co] and years of exposure shows the relationship of both parameters to the CSIS.)

Results:

- Report demographics of cohort - age, gender, implant types. Lines 139-156 and Tables 2&3.

- Present urine cobalt levels summary statistics before analyzing by cobaltism symptoms. Reordered as requested, Lines 158-164 and Tables 2&3.

- Use tables/figures to clearly show symptom scores for each domain by cobalturic vs non-cobalturic groups. See Tables 2, 4, 5, 6, and Figures 1&2.

Discussion:

- Note limitations including single-center cohort, selection bias, lack of universal cobaltism criteria. See Lines 291-337

- Compare associations found to prior studies linking urine cobalt to clinical effects. See Lines 206-216

- line 175, alternative causes of vertigo should be discussed as drugs or viral infections. please Cite doi: 10.1177/0960327111414280 and doi:10.3389/fmed.2021.790931. See Lines 242-247, and added references:

40.	Agrup C, Gleeson M, Rudge P. The inner ear and the neurologist. Journal of Neurology, Neurosurgery & Psychiatry. 2007;78(2):114-22.

41.	Di Mauro P, La Mantia I, Cocuzza S, Sciancalepore PI, Rasà D, Maniaci A, et al. Acute vertigo after COVID-19 vaccination: case series and literature review. Frontiers in medicine. 2022;8:790931.

- Discuss need for multi-center studies to validate questionnaire, urine thresholds. See lines 291-337.

- Suggest future research on treatment effects tied to urine cobalt reductions. See lines 328-337.
- Avoid overstating the conclusions - cohort limitations preclude definitive conclusions. The adjectives “likely” and “apparent” liberally applied.

Reviewer #2: I read with interest the manuscript by Tower et al. The paper is original and sound. However, there are some issues that need to be addressed with a revision:
- I could not find the abstract anywhere in the manuscript. Please provide it. See lines 1-28.

- Line 50-51. Authors should also cite hearing loss and delirium as possible adjunct misdiagnosis of cobaltism (doi: 10.1177/0960327111414280 - doi: 10.3390/jcm12020435). Please briefly discuss and add these 2 references. See lines 61-73 and 124-129

Added references

33.	Asker S, Asker M, Yeltekin AC, Aslan M, Demir H. Serum levels of trace minerals and heavy metals in severe obstructive sleep apnea patients: correlates and clinical implications. Sleep and Breathing. 2015;19:547-52.

48.	Agrup C, Gleeson M, Rudge P. The inner ear and the neurologist. Journal of Neurology, Neurosurgery & Psychiatry. 2007;78(2):114-22.

49.	Di Mauro P, La Mantia I, Cocuzza S, Sciancalepore PI, Rasà D, Maniaci A, et al. Acute vertigo after COVID-19 vaccination: case series and literature review. Frontiers in medicine. 2022;8:790931.

- Line 58-64. These sentences summarize both methods and results, and should therefore be not included in the introduction section. This content moved to methods.

- Please provide ethical committee approval or waiver code and date of approval. See lines 81-86.

- How did authors retrieve data from patients with dementia? Were they excluded? Please specify. See line 86.

- Please provide the main investigated outcome in the methods section. See lines 90-93.

- Lack of data on cardiovascular function of the patients enrolled should be highlighted as a further possible limitation of the study and direction of future research, as echocardiography is nowadays a feasible bedside tool to investigate systolic and diastolic function of hospitalized patients (doi: 10.2106/JBJS.16.00743- doi: 10.1007/s12630-022-02225-0 - [1]. Please discuss and add these 3 references. See lines 323-326, added references:

52.	Berber R, Abdel-Gadir A, Rosmini S, Captur G, Nordin S, Culotta V, et al. Assessing for cardiotoxicity from metal-on-metal hip implants with advanced multimodality imaging techniques. The Journal of bone and joint surgery American volume. 2017;99(21):1827.

53.	Sanfilippo F, La Via L, Flower L, Madhivathanan P, Astuto M. The value of subcostal echocardiographic assessment, and directions for future research. Canadian Journal of Anesthesia/Journal canadien d'anesthésie. 2022;69(5):676-7.
